# BMAA Inhibits Nitrogen Fixation in the Cyanobacterium *Nostoc* sp. PCC 7120

**DOI:** 10.3390/md11083091

**Published:** 2013-08-21

**Authors:** Lotta Berntzon, Sven Erasmie, Narin Celepli, Johan Eriksson, Ulla Rasmussen, Birgitta Bergman

**Affiliations:** Department of Ecology, Environment and Plant Sciences, Stockholm University, Stockholm S-10691, Sweden; E-Mails: sven.erasmie@karolinska.se (S.E.); narin.celepli@su.se (N.C.); johan.eriksson@su.se (J.E.); ulla.rasmussen@su.se (U.R.); birgitta.bergman@su.se (B.B.)

**Keywords:** cyanobacteria, toxin, BMAA, *Nostoc* sp. PCC 7120, nitrogen fixation, nitrogenase activity

## Abstract

Cyanobacteria produce a range of secondary metabolites, one being the neurotoxic non-protein amino acid β-*N*-methylamino-L-alanine (BMAA), proposed to be a causative agent of human neurodegeneration. As for most cyanotoxins, the function of BMAA in cyanobacteria is unknown. Here, we examined the effects of BMAA on the physiology of the filamentous nitrogen-fixing cyanobacterium *Nostoc* sp. PCC 7120. Our data show that exogenously applied BMAA rapidly inhibits nitrogenase activity (acetylene reduction assay), even at micromolar concentrations, and that the inhibition was considerably more severe than that induced by combined nitrogen sources and most other amino acids. BMAA also caused growth arrest and massive cellular glycogen accumulation, as observed by electron microscopy. With nitrogen fixation being a process highly sensitive to oxygen species we propose that the BMAA effects found here may be related to the production of reactive oxygen species, as reported for other organisms.

## 1. Introduction

Cyanobacteria produce a vast range of secondary metabolites, and yet the functions of these compounds remain largely unknown. Many are uncharacterized, but several have been classified as toxins, due to their severely negative impact on eukaryotic organisms, such as domesticated and wild animals, and in some rare cases even on humans [1]. Although toxin-producing cyanobacteria are globally widespread in both aquatic and terrestrial environments, the biosynthesis of the cyanotoxins is restricted to a limited number of genera within the cyanobacterial phylum. Some of the best-known examples of toxins produced by cyanobacteria are microcystins (*Microcystis* spp. and *Anabaena* spp.), nodularin (*Nodularia spumigena*), and anatoxin-a (*Anabaena flos-aquae* and *Phormidium* spp.) [1,2]. As toxin-producing cyanobacteria often form massive surface accumulations, known as blooms, in aquatic environments, including touristic and recreational coastal areas, the production of toxins is considered a nuisance and a potential health issue. Genome sequencing of toxic bloom-forming cyanobacteria has revealed an array of biosynthetic gene clusters involved in toxin production and has facilitated a deeper understanding of the role and regulation of such bioactive compounds [3,4]. 

The recently described cyanobacterial toxin, β-*N*-methylamino-L-alanine (BMAA), shows some characteristics that clearly differ from those of other cyanotoxins. For instance, BMAA is a small non-protein amino acid that appears to be synthesized by the entire cyanobacterial phylum [5,6], suggesting that BMAA is a fundamental cellular metabolite in cyanobacteria. Additionally, BMAA is a slow-acting toxin [7,8], and, in contrast to other cyanotoxins, not known to elicit any acute effects [9]. BMAA was, early on, implicated as the causative agent of amyotrophic lateral sclerosis/parkinsonism dementia complex (ALS/PDC) and later as one of the potential long-sought-after environmental factor eliciting human neurodegeneration in general (e.g., ALS, Alzheimer’s, and Parkinson’s disease) [10,11]. Negative impacts of BMAA on aquatic organisms (below micromolar concentrations) have also been observed [12,13,14,15,16], implying a significance of this toxin in natural ecosystems.

To date, BMAA has been detected in numerous cyanobacterial field samples [5,6,17]. For instance, the presence of BMAA was discovered in a natural cyanobacterial population forming large surface scums (blooms) in a temperate aquatic ecosystem, the Baltic Sea, surrounded by nine countries and ninety million inhabitants [18]. The study showed that BMAA was produced throughout the cyanobacterial bloom-forming season at low but detectable levels. Moreover, considerably elevated BMAA concentrations were found in organisms of higher trophic levels (*i.e.*, zooplankton, mollusks, and fish) that are dependent on cyanobacterial-based food webs, thus revealing a natural route for transfer through an aquatic ecosystem and a human exposure via food intake [18]. 

Reported levels of BMAA in cyanobacteria range from non-detectable to thousands of micrograms per gram dry weight [5,6,18,19,20], indicating that cyanobacterial BMAA production may vary considerably under both natural and culture conditions. This was further supported by the induction of BMAA biosynthesis in two unicellular cyanobacteria during nitrogen starvation [21]. Moreover, as BMAA has not been detected in surrounding waters despite high intracellular BMAA levels in examined cyanobacteria [22], cellular export may not take place and BMAA might be predominantly released at cell lysis, as shown for other cyanotoxins [23,24]. These factors contribute to the difficulties of estimating potential concentrations of BMAA in aquatic environments. 

As cyanobacteria are ancient organisms dating back at least three billion years, it is reasonable to assume that BMAA has an important role in the physiology of cyanobacteria and that any connection to human neurodegeneration is merely coincidental. 

However, the understanding of the nature and significance of BMAA biosynthesis and regulation in cyanobacteria is today limited, even though this is ultimately crucial for preventing human exposure and the spread of toxic BMAA in nature. As micromolar concentrations of BMAA (4.2–42 µM) reduced growth of the cyanobacterium *Synechocystis* PCC 6803 [25], we decided to investigate the role of BMAA in *Nostoc* sp. PCC 7120, a cyanobacterium of the same nitrogen-fixing type as the bloom-forming species [26] examined earlier [18]. Nitrogen fixation was severely inhibited by BMAA and the data are discussed in relation to cyanobacterial bloom demise.

## 2. Results

The cyanobacterial genus *Nostoc* occurs throughout terrestrial and aquatic habitats world-wide, and belongs to a filamentous cyanobacterial phylogenetic clade capable of fixing atmospheric dinitrogen in heterocysts (Section IV; [27]). The sequenced and well-studied strain *Nostoc* sp. PCC 7120 (also known as *Anabaena* sp. PCC 7120, hereafter *Nostoc* 7120) selected as the test organism was previously reported to produce BMAA [5], which was also verified for our strain using LC-MS/MS analysis (See Supplementary Figure S1). Thus, BMAA appears to be a natural cellular constituent of this strain. The applied concentrations of BMAA in this study are in line with a previous exposure experiment for cyanobacteria [25].

### 2.1. ^14^C-BMAA Uptake

**Figure 1:**
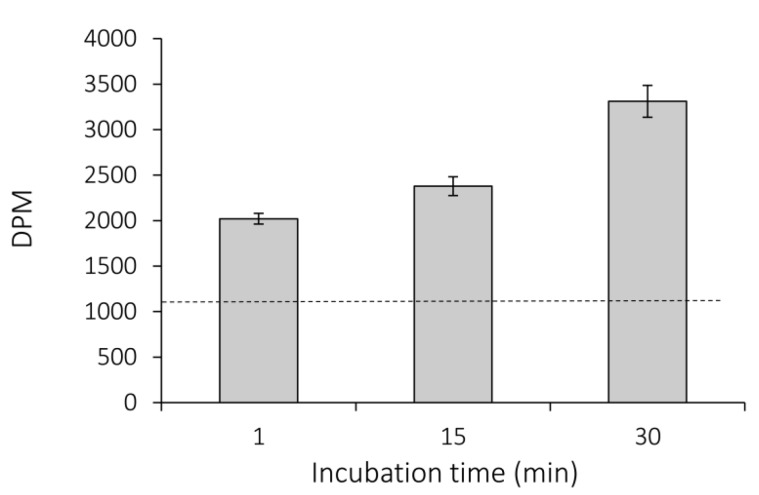
Uptake of ^14^C-BMAA by *Nostoc* 7120. Average uptake of 10 µM ^14^C-BMAA measured as decay per minute (DPM) is given for various time points. Radioactive levels above those of the blank (boiled cells exposed for 30 min, dashed line ± SE 58.9) are interpreted to represent cellular BMAA uptake. ±SE; *n* = 3.

The ability of *Nostoc* 7120 to take up exogenously applied BMAA was first examined by monitoring the uptake of 10 µM ^14^C-labelled BMAA, added to actively growing cultures. To estimate any unspecific association to cell constituents, an equal concentration of ^14^C-BMAA was added to boiled cells (blank). As shown in Figure 1, there was already an uptake of ^14^C-BMAA at one minute after exposure, compared to that observed for the boiled cells, as verified by a two-sample *t*-test (*p* < 0.001). Moreover, we demonstrated a positive correlation between time of exposure and ^14^C-BMAA uptake, as verified by linear regression analysis (*R*^2^-value = 0.87, *p* < 0.001).

### 2.2. Effects of BMAA on Nitrogenase Activity

As nitrogen fixation is a fundamental process in many aquatic bloom-forming cyanobacteria, such as those in the brackish Baltic Sea [28] and in tropical oceans [29], we decided to examine the effect of BMAA on this process. To induce nitrogen fixation, *Nostoc* 7120 cultures were grown in BG11_0_ medium [27]. Acetylene reduction assay-gas chromatography (ARA) was used to examine the effects of BMAA on the activity of nitrogenase, the enzyme complex catalyzing the reduction of dinitrogen to NH_4_^+^. The BMAA structural isomer 2,3-diaminobutyric acid (DAB) and glutamate (Glu), which structurally resemble the carbamate form of BMAA, were included for comparison, as were the highly potent inhibitors of nitrogenase activity, ammonium (NH_4_^+^) [30] and cysteine (Cys) [31]. The amino acids were of the l-form and added at 20 µM, while NH_4_^+^ was added at a 250-fold higher concentration (5 mM), which was known to efficiently suppress nitrogenase activity. 

**Figure 2:**
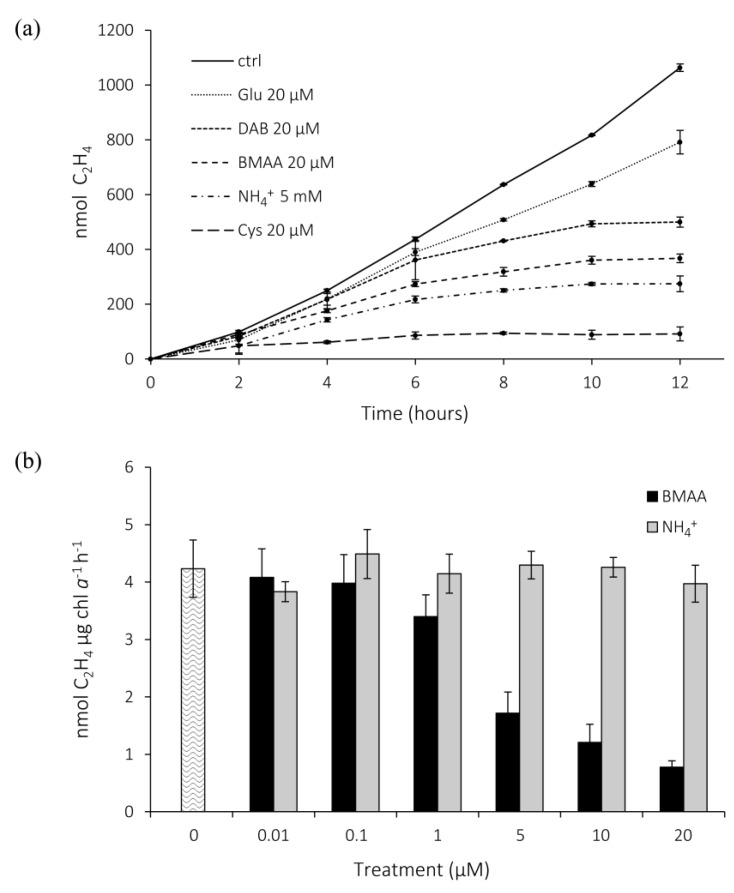
Nitrogenase activity in *Nostoc* 7120 upon the addition of BMAA and various nitrogen sources. (**a**) Accumulated ethylene production (nmol C_2_H_4_) after the addition of 20 µM BMAA, DAB, Cys, or Glu, or 5 mM NH_4_^+^. The graph displays mean values from one of the two experiments ± SE; *n* = 3. (**b**) Ethylene production (nmol C_2_H_4_ µg chl *a*^−1^ h^−1^) after addition of BMAA or NH_4_^+^ at various concentrations to the BG11_0_-grown *Nostoc* 7120 cells (20-h treatment). The bars denote mean values of three independent experiments ± SE; *n* = 3.

As seen in Figure 2a, BMAA reduced nitrogenase activity compared to the control (*p* < 0.05; two-way ANOVA and Tukey’s HSD test of the difference of average slopes between two and 10 h; two independent experiments). The inhibitory effects of BMAA did not differ significantly from those of NH_4_^+^ (*p* = 0.97) added at 5 mM. Glu and DAB slightly reduced the activity, although not significantly compared to the control (*p* = 0.70 and 0.20, respectively). On the other hand, Cys reduced the nitrogenase activity (difference from control; *p* < 0.001) to an even higher, although not statistically significant, extent than BMAA (difference from BMAA; *p* = 0.22).

Next, we examined the effective concentrations of BMAA on nitrogenase activity. BMAA negatively affected nitrogenase activity at concentrations of 1–5 µM, while no clear inhibitory effect was observed for NH_4_^+^ at any of the tested concentrations (Figure 2b) (*p* < 0.01; paired *t*-test of the difference of average slopes for the two treatments; three independent experiments).

Effects of BMAA on Nitrogenase Activity Compared to Other Amino Acids and Nitrogen Sources 

**Figure 3:**
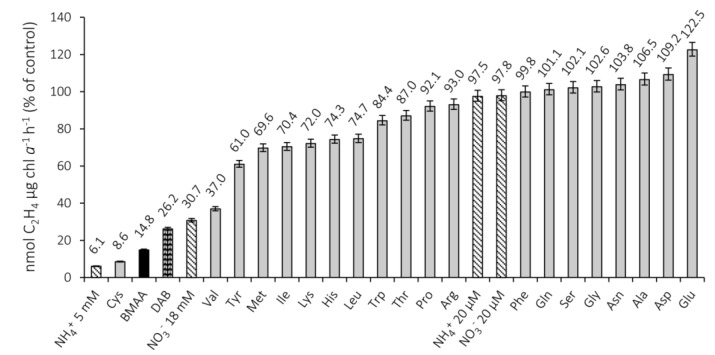
Nitrogenase activity in the presence of BMAA, amino acids, or combined nitrogen sources. Nitrogenase activity expressed as a percentage of the control activity, with no compounds added to the standard growth medium lacking nitrate (BG11_0_), after a 20-h treatment with BMAA, DAB, or the standard proteinogenic amino acids at 20 µM. NO_3_^−^ and NH_4_^+^ were examined at both low (20 µM) and high (18 mM or 5 mM, respectively) concentrations added. The bars denote the mean values calculated from two independent experiments with error bars showing the 95% confidence interval.

We next compared the effects of BMAA on nitrogenase activity with those of the 20 standard amino acids as well as DAB (all in the l-form) at a concentration of 20 µM (Figure 3). The combined nitrogen sources, ammonium (NH_4_^+^) and nitrate (NO_3_^−^) were also added at 20 µM and at concentrations typical of cyanobacterial growth medium, *i.e.*, 5 mM NH_4_^+^ and 18 mM NO_3_^−^ (including the approximately 20 µM NH_4_^+^ in the BG11_0_ medium). With the exception of Cys, BMAA was a more potent inhibitor than all of the other amino acids tested, with only 14.8% of the control nitrogenase activity remaining after a 20-h treatment. Cys severely inhibited the nitrogenase activity, with just 8.6% of the activity remaining at 20 h. DAB also had a pronounced effect, with only 26.2% of the control nitrogenase activity remaining after a 20-h treatment. The decrease in nitrogenase activity upon the addition of BMAA, DAB, and Cys was significant (unadjusted *p* < 0.001 for all three; two-way ANOVA and *t*-test adjusted with Bonferroni correction). In contrast, treatment with 20 µM NO_3_^−^ or NH_4_^+^ did not affect nitrogenase activity to any considerable amount, while treatment with 18 mM and 5 mM, respectively, reduced the activity to 30.7% (NO_3_^−^) and 6.1% (NH_4_^+^) of control activity (unadjusted *p* < 0.001 for both NO_3_^−^ and NH_4_^+^). Val lowered the nitrogenase activity to 37.0% of the control activity.

### 2.3. Transmission Electron Microscopy (TEM)

**Figure 4:**
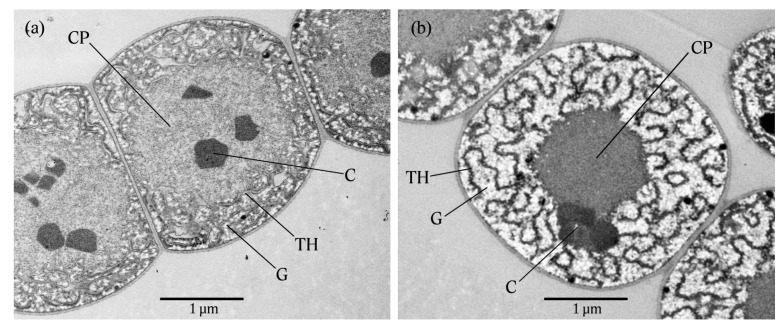
TEM micrographs illustrating the cellular response of Nostoc 7120 to BMAA. Vegetative Nostoc 7120 cells (**a**) in the absence of BMAA (control) and (**b**) subjected to a 24-h treatment with 20 µM BMAA. Note the accumulation of electron-transparent glycogen granules (carbon storage) between the thylakoid membranes. G, glycogen; C, carboxysomes; TH, thylakoid membranes; CP, centroplasm.

The cellular consequences of externally added BMAA were examined in *Nostoc* 7120 subjected to a 24-h treatment with 20 µM BMAA using transmission electron microcopy (TEM). Examination of ultrathin sections showed that the vegetative cells grown in the absence of BMAA displayed organized photosynthetic thylakoid membranes, enclosing moderate levels of glycogen (carbon storage), as well as groups of carboxysomes, which contain RuBisC/O, in the large centroplasm (Figure 4a). The most striking feature of BMAA-treated cells is the noticeable accumulation of electron-transparent glycogen granules between the thylakoid membranes (Figure 4b), a finding suggesting nitrogen depletion.

### 2.4. BMAA—Long Term Effects

**Figure 5:**
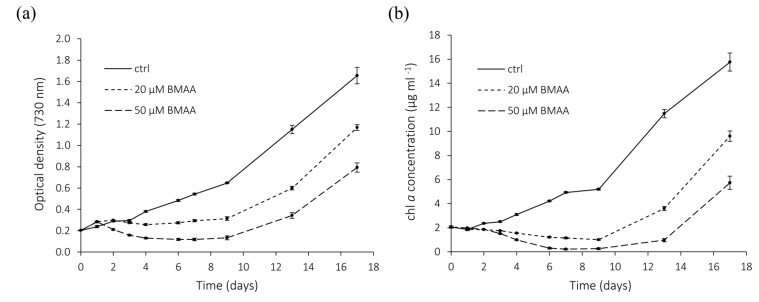
Effects of BMAA on the growth of *Nostoc* 7120. Cell cultures were monitored for 18 days in the absence (ctrl) or presence of 20 or 50 µM BMAA. The graphs illustrate mean values ± SE; *n* = 5. (**a**) Optical density (OD) at 730 nm. (**b**) Chl *a* concentration (µg/mL).

To assess the long-term effects of BMAA on *Nostoc* 7120 growth, the optical density (OD) at 730 nm, which serves as an indicator of cell density, and chl *a* concentration were monitored for 18 days. Figure 5 shows that both 20 and 50 µM BMAA had a negative, concentration-dependent impact on growth during the first half of the experiment, starting approximately after two days of treatment. The effects of 20 and 50 µM BMAA differed significantly from each other and from the control (*p* < 0.001 for both OD and chl *a* experiments; one-way ANOVA and Tukey’s HSD test of difference of the average slopes at days two to seven). The characteristic blue-green pigmentation shifted towards yellow-green for BMAA-treated cells (data not shown). This chlorotic state may be attributed to the breakdown of chl *a* and the pigment-rich phycobilisomes [32]. The growth inhibition persisted for approximately seven days, at which point the cells started to recover and eventually regained a growth rate similar to that of the control.

Because *Nostoc* 7120 cells could resume growth after seven days of BMAA treatment, we examined whether this was caused by cellular effects or by degradation/alteration of the BMAA molecule in the medium over time. Hence, 30 µM BMAA was maintained in BG11_0_ growth medium without any added cells for up to four weeks, and subsequently tested for its capacity to inhibit the nitrogenase activity of *Nostoc* 7120 in a 24-h treatment. As shown in Table 1, there was a clear and significant reduction in the nitrogenase activity of cultures incubated with fresh BMAA compared to the activity of cultures without BMAA (*t*-test; *p* < 0.001). The nitrogenase activity of *Nostoc* 7120 cells treated with BMAA kept in cell-free BG11_0_ medium for four weeks prior to the experiment, was reduced to the same extent as the cells incubated with fresh BMAA (not preincubated in the BG11_0_ medium) (*p* = 0.32, *t*-test). These data suggest that BMAA was not spontaneously degraded or significantly altered with time, indicating that the recovery observed after seven days of treatment (Figure 5) is likely due to metabolic turnover and/or other cellular effects in *Nostoc* 7120.

**Table 1:** Capacity of BMAA to reduce nitrogenase activity in *Nostoc* 7120 following maintenance in cell-free BG11_0_ medium. Nitrogenase activity of *Nostoc* 7120 after a 24-h incubation with fresh BMAA (30 µM) or with BMAA (30 µM) first kept in cell-free BG11_0_ medium for the indicated time periods.

BMAA treatment	Nitrogenase activity (nmol ethylene µg chl *a*^−1^ h^−1^)	±SE (*n* = 3)
No BMAA added	5.9	0.240
Fresh BMAA added	0.2 *	0.033
1 week	0.2	0.082
2 weeks	0.4	0.038
3 weeks	0.4	0.032
4 weeks	0.2 (ns)	0.029

Week(s) = Time of maintaining BMAA in BG11_0_ medium prior to the addition of *Nostoc* 7120 cells; * Significantly different from no BMAA added (*p* < 0.001); ns = Not significantly different from fresh BMAA added (*p* = 0.32).

## 3. Discussion

Since BMAA may be an environmental factor triggering human neurodegeneration, it is of great importance to elucidate the role of BMAA not only in this human-related context, but also to better comprehend the nature and significance of BMAA in cyanobacteria. To expand our knowledge, we analyzed the physiological and morphological consequences of externally adding BMAA to the nitrogen-fixing cyanobacterium, *Nostoc* 7120. Together, our data show that this cyanobacterium synthesizes BMAA and, when applied externally, BMAA is taken up in a fast and concentration-dependent manner. The latter observation is in accordance with data for BMAA uptake by the unicellular non-nitrogen-fixing cyanobacterium *Synechocystis* sp. PCC 6803 [25]. *Nostoc* 7120 has been reported to take up amino acids using mainly three systems, N-I, N-II, and Bgt [33,34]. Being predominantly uncharged under physiological conditions [35], BMAA uptake is postulated to occur via the N-I or N-II system. Our data also suggest that BMAA is not degraded extracellularly, but is stably maintained over the time period of the experiments, indicated by its preserved capacity of reducing the nitrogenase activity of *Nostoc* 7120 even when kept in cell-free medium for several weeks. Notably, BMAA is known to react with bicarbonate ions at physiological concentrations in eukaryotic cell cultures, forming a stable β-carbamate with structural similarities to glutamate [36,37,38]. If, and to what extent, this reaction takes place in the carbon containing (BG11_0_) medium used here, would have to be experimentally tested. The onset of the inhibitory effects of BMAA on nitrogen fixation was rapid (within about 2–4 h) and apparent at micromolar concentrations. BMAA was furthermore revealed to be a several-fold stronger inhibitor of nitrogen fixation than NH_4_^+^ and NO_3_^−^ when added at the same concentrations. In contrast to the non-protein amino acid BMAA, all proteinogenic amino acids showed limited inhibitory effects on nitrogenase activity, except for Cys, which drastically decreased nitrogenase activity, as did DAB, a structural isomer of BMAA. 

BMAA also markedly retarded growth within days of application, suggesting that BMAA was not used as a source of nitrogen in spite of its composition (which includes two nitrogen atoms), but must affect the nitrogenase activity by some other mechanism. As the ultrastructural analysis revealed that externally added BMAA caused massive accumulation of the cellular carbon-storage molecule glycogen, this rather suggests a cellular nitrogen deficiency and an increased C:N ratio [39,40]. This conclusion was here also supported by signs of chlorosis in the BMAA-treated cultures. Similarly, growth retardation and chlorosis was observed in *Synechocystis* PCC 6803 during BMAA exposure [25]. Interestingly, BMAA biosynthesis was also recently reported to be enhanced in the non-nitrogen-fixing cyanobacteria *Microcystis* PCC 7806 and *Synechocystis* J341 as a response to nitrogen depletion [21]. Externally added BMAA may therefore enhance the intracellular levels of BMAA in *Nostoc* 7120 in two ways, by (1) allowing its uptake and, (2) stimulating the cellular biosynthesis of BMAA due to the nitrogen depletion elicited. Together, these data show that BMAA does not limit nitrogenase activity by acting as a nitrogen source, but rather that BMAA functions as a potent inhibitor of nitrogenase activity and/or of related physiological processes. Notably, BMAA has been shown to inhibit the synthesis and/or stimulate the degradation of glutamine in rat tissues [35], and to induce glutamate release by acting on system Xc^−^ (cystine/glutamate antiporter) in cortical cell cultures (mice) [41]. We propose that similar mechanisms may be operative in cyanobacteria, as such mechanisms would have detrimental effects on their nitrogen-fixing physiology. Glutamate is the acceptor molecule for the NH_4_^+^ produced in the heterocysts during nitrogen fixation, which leads to the formation of glutamine that in turn is exported to the vegetative cells, where it acts as the precursor of glutamate and hence of all other amino acids. If BMAA would reduce glutamine levels or stimulate glutamate release from the cells, also in cyanobacteria, this could rapidly cause a buildup of intracellular NH_4_^+^, with a subsequent inhibition of nitrogenase activity as recorded here in *Nostoc* 7120.

Moreover, the β-carbamate of BMAA acts on glutamate receptors in eukaryotic cells [42,43,44,45,46], causing an increase in cellular Ca^2+^ levels which elicits oxidative stress via the release of reactive oxygen species (ROS) from mitochondria [42,46]. BMAA also induces oxidative stress by inhibiting cystine (disulfide of two cysteine) uptake via system Xc^−^, causing depletion of glutathione, a cellular metabolite protecting against oxidative stress [41]. Although most studies on the toxic cellular mechanisms of BMAA have been performed on rodent neuronal cells [47], recent work suggests that BMAA may also induce oxidative stress in aquatic plants [15,16]. Likely, similar cellular mechanisms may be operative in cyanobacteria. For instance, the rapid reduction in nitrogenase activity found in our analyses appears to mimic that caused by O_2_. In cyanobacteria, both the transcription and translation of the nitrogenase structural genes (*nif*KDH), and in particular the biosynthesis of the Fe-protein (encoded by *nif*H), are extremely sensitive to enhanced O_2_ levels, as is nitrogenase activity. ROS may be produced in cyanobacteria via their oxygenic photosynthesis [48] and negatively influence nitrogen fixation activities. Considering that cyanobacteria, in spite of their prokaryotic nature, also possess the glutamate receptor GluR0 [49], and that glutathione acts as a protectant against cellular oxidative stress in cyanobacteria [50], enhanced concentrations of BMAA apparently causing nitrogen starvation, as shown here, may lead to cellular stresses and ROS production.

The fact that the inhibitory effect of BMAA on growth eventually ceased (after ~7 days), allowing the cells to recover and regain their pigmentation, implies that BMAA is either metabolized by the cyanobacterium or degraded. Indeed, BMAA might be metabolized or modified into harmless constituents, or some other BMAA protection mechanism(s) may be induced, such as the incorporation of BMAA into proteins, as shown recently for human cells [51]. We excluded the possibility that BMAA is externally degraded/altered by showing that BMAA keeps its ability to reduce nitrogenase activity in *Nostoc* PCC 7120 even after several weeks of maintenance in cell-free growth medium. The stability of BMAA in liquid medium has also previously been reported [14]. 

Our comparison of the effects of BMAA on nitrogenase activity with those of the 20 proteinogenic amino acids identified Cys as an amino acid with a similar capacity to inhibit nitrogenase activity as BMAA. Interestingly, Cys has been reported to have toxic effects similar to those of BMAA, and the toxicity is enhanced in the presence of bicarbonate [52]. DAB, a neurotoxic structural isomer of BMAA [53], also had similar (albeit somewhat lower) reducing effects on the nitrogenase activity of *Nostoc* 7120. Hence, BMAA, DAB, and Cys, all of which are known to have toxic effects on eukaryotic cells, are effective inhibitors of nitrogenase activity in a prokaryotic cyanobacterium. The negative effects of Cys on cyanobacterial nitrogenase activity have been observed previously [31] and it is now of interest to determine whether Cys and BMAA (DAB) use the same toxicity mechanisms in cyanobacteria.

Remaining questions relate to why cyanobacteria produce an autotoxic compound, as shown here, and the significance of our findings for cyanobacteria in nature. Our data suggest that BMAA is not harmful to cyanobacteria at low intracellular concentrations but inhibits a key physiological process at micromolar (and above) cellular concentrations. This situation may arise within blooms as the population increase and some cells start to lyse, due to for instance shading and nutrient limitation. Above certain BMAA thresh-hold levels (e.g., 5–10 µM) processes such as ROS production (as in eukaryotes [41,42,46]) and programmed cell death (PCD) may be elicited. This in turn may lead to cell lysis and subsequent bloom collapse. Indeed, PCD has been proposed to cause the demise of phytoplankton blooms including blooms of tropical cyanobacteria [54,55,56,57]. In addition, metacaspases (caspase homologs) are common among bloom-forming cyanobacteria [58]. The involvement of BMAA, alone or in concert with other signaling molecules, in such a cascade is a hypothesis now worth testing experimentally. 

## 4. Experimental Section

### 4.1. Chemicals

l-BMAA (l-BMAA hydrochloride B-107) and the proteinogenic amino acids (l-form) were from Sigma-Aldrich (Steinheim, Germany). l-2-4-diaminobutyric acid hydrochloride (l-DAB) was from Fluka (#32830; Fluka, Buchs, Switzerland) and l-^14^C-BMAA (β-*N*-methylamino-l-alanine (*N*-methyl-14C) ARC 3042) from Biotrend (St. Louis, MO, USA). Stock solutions of the amino acids were prepared in water (MS-grade) or 0.1 M HCl in the case of tyrosine (Tyr).

### 4.2. Organisms and Cultivation

Batch cultures of *Nostoc* sp. PCC 7120 were grown on a shaker in continuous light (~20 µmol m^−2^ s^−1^), at 25 °C in BG11_0_ medium (nitrate-free to elicit the nitrogen fixation process) [27] buffered with 7–10 mM HEPES. All experiments were conducted with cultures diluted to an initial optical density of 0.2 (±0.02) at 730 nm (OD_730_) (corresponding to ~2.4 ± 0.7 µg chl *a*/mL) if not otherwise stated. These specified growth conditions were used for all experiments.

### 4.3. Growth

Growth of *Nostoc* 7120 was monitored as OD_730_ and chl *a* concentrations for up to 18 days for non-exposed cultures and for those exposed to 20 or 50 µM BMAA. For each treatment, five biological replicates of 20-mL cultures in 100-mL Erlenmeyer flasks were performed, as were technical triplicates for all chl *a* and OD measurements. OD_730_ was used as an estimate of cell density (turbidity). The absorbance of 1-mL cell cultures was measured in an Ultrospec 3000 spectrophotometer (Pharmacia Biotech, Cambridge, England). All samples were properly mixed before measurement, and when necessary also diluted or concentrated to fit within the linear range. For chl *a* analysis, 1 mL *Nostoc* 7120 culture was collected and centrifuged at 27,770× *g* (Hettich EBA12 UNIVERSAL 16/16R centrifuge, Andreas Hettich GmbH & Co.KG, Tuttingen, Germany). The supernatant was discarded and the pellet was stored at −20 °C prior to analysis. For extraction of chl *a*, the pellet was dissolved in 1 mL 90% MeOH and incubated at +4 °C in darkness for 3 h. The sample was subsequently centrifuged and the supernatant was used for measuring OD_665_. The chl *a* concentrations were calculated according to Talling and Driver [59] using 13.9 as the specific absorption coefficient for chl *a* in 90% MeOH, and with correction for turbidity at 750 nm.

### 4.4. ^14^C-BMAA Uptake

The ^14^C-BMAA uptake assay was performed according to Montesinos *et al.* [33] with minor modifications. Briefly, 100 µL of 110 µM L-^14^C-BMAA (Biotrend) was added to 1 mL *Nostoc* 7120 culture of OD_730_ = 0.63 (corresponding to 5.2 µg chl *a*). Thus, the total reaction volume was 1.1 mL with a final ^14^C-BMAA concentration of 10 µM. The mix was incubated under standard growth conditions (see Section 4.2) and collected in triplicate for analysis after 1, 15, or 30 min. The cells were collected by filtration on 13-mm diameter filters with a 3-µm pore size (Millipore, White SSWP, Piscataway, NJ, USA), and washed twice with 20 mL of 25 mM *N*-tris(hydroxymethyl)-methylglycine (Tricine)-NaOH buffer (0.25 M NaCl, pH 8.1). Cells boiled for 15 min before addition of ^14^C-BMAA were used as a blank to reveal unspecific binding to cell material and were incubated for 30 min with the same amount of ^14^C-BMAA as the experimental samples. The filters were subsequently submerged in 10 mL scintillation fluid and radioactivity was measured (1409 Liquid Scintillation counter, Wallac OY, Turku, Finland). 

### 4.5. Nitrogenase Activity

The nitrogenase activity was determined using the acetylene reduction assay (ARA) coupled to gas chromatography and calculated as the amount of ethylene (C_2_H_4_) µg chl *a*^−1^ h^−1^ produced according to Capone and Montoya [60]. A 100-ppm ethylene standard was used to calculate the ethylene concentrations. Aliquots of *Nostoc* 7120 were placed in glass vials sealed with rubber septa. Acetylene gas (C_2_H_2_) replaced 10% of the gas phase (air) and the vials were subsequently incubated for 2 h under standard growth conditions (see Section 4.2). A portion of the gas phase was analyzed for ethylene content using gas chromatography with a flame ionization detector (GC-FID) (Shimadzu Model GC-8AIF, Kyoto, Japan) equipped with a Porapak N 80/100 mesh column (Shimadzu, Kyoto, Japan), using N_2_ as a carrier gas. Peaks were displayed with the software Chromatography Station for Windows (CSW) version 1.7 (DataApex Ltd, Prague, The Czech Republic). Vials with BG11_0_ medium alone plus acetylene, or cell cultures with no added acetylene were used as negative controls. The cell culture alone did not give rise to any ethylene production, whereas medium incubated with acetylene showed a small ethylene peak, which was considered to be the baseline level and was subsequently subtracted from the ethylene peak area of the experimental samples.

#### 4.5.1. Accumulated Nitrogenase Activity

A volume of 20 mL of *Nostoc* 7120 culture added to 100 mL Erlenmeyer flasks fitted with rubber stoppers was mixed with 20 µM BMAA/DAB/Cys or 5 mM NH_4_^+^ and compared to untreated controls (triplicate flasks for all). At the start of the experiment, 10 mL of the gas phase was replaced with acetylene gas. Every second hour during the 12-h study, 0.5 mL gas from each flask was withdrawn and assayed for ethylene production. Two independent experiments of identical set-up were conducted.

#### 4.5.2. Concentration-Dependent Effects of BMAA and NH4+ on Nitrogenase Activity

BMAA was added to 10 mL *Nostoc* 7120 culture in 50 mL Erlenmeyer flasks to a final concentration of 0.01, 0.1, 1, 5, 10, or 20 µM and incubated for 20 h. NH_4_^+^ was similarly added in the same amounts as BMAA, in addition to the approximately 20 µM already present in the BG11_0_ medium [27]. All treatments, as well as a control (without any compound added), were performed in triplicate. After incubation, 1.5 mL of each culture was transferred to 4.7 mL glass vials with rubber septa and incubated for 2 h with 0.4 mL acetylene. Ethylene content was analyzed in 0.5 mL of the gas phase and normalized against chl *a* content. The experiment was repeated three times.

#### 4.5.3. Effect of BMAA and Other Amino Acids and Nitrogen Sources on Nitrogenase Activity

Ten milliliters of *Nostoc* 7120 culture was added to 50 mL Erlenmeyer flasks supplemented with 20 µM BMAA, DAB, or one of the 20 proteinogenic amino acids. Ammonium (NH4+) and nitrate (NO3−) were supplemented at the same concentration as the amino acids (20 µM), but also at 5 mM for NH4+ and 18 mM for NO3−, although the total concentration of NH4+ were higher (see Section 4.5.2). All treatments, including an untreated control, were examined in triplicate and the analysis was performed as described in Section 4.5.2. Two independent experiments were performed.

#### 4.5.4. Capacity of BMAA to Reduce Nitrogenase Activity in *Nostoc* 7120 Following Maintenance in Cell-Free BG11_0_ Medium

BMAA was added at a concentration of 30 µM to 30 mL BG11_0_ medium (in 100 mL Erlenmeyer flasks) and kept for 1, 2, 3, or 4 weeks in standard growth conditions (see Section 4.2) before *Nostoc* 7120 cells were added to the medium. Other cultures were treated with fresh BMAA, whilst the control cultures had no external BMAA added. The cells of the original culture were pelleted by centrifugation (1470× *g*) and equally portioned out to the flasks containing BG11_0_ medium with BMAA. The procedure resulted in all treatments containing 30 mL *Nostoc* 7120 culture of OD_730_ = 0.2. Triplicate samples were incubated for 24 h followed by analysis of nitrogenase activity (*n* = 3; Section 4.5.2).

### 4.6. Transmission Electron Microscopy (TEM)

The embedding procedure of Lundgren *et al.* [61] was slightly modified and performed as follows. Sixty milliliters of *Nostoc* 7120 culture, with or without 20 µM BMAA, was grown in 200 mL Erlenmeyer flasks. The cultures were incubated for 24 h, and 9 mL of culture was then immediately fixed in 1 mL glutaraldehyde (25%, EM grade, TAAB Laboratories Equipment Ltd., Aldermaston, Berks, UK), giving a final concentration of 2.5% (vol/vol) glutaraldehyde. The fixed samples were stored at 4 °C in darkness before being embedded. The glutaraldehyde was discarded after centrifugation (2 min at 2630× *g* at 4 °C), and the fixed cells were subsequently washed three times in 9.6 mM phosphate buffer (11.5 g Na_2_HPO_4_ and 2 g KH_2_PO_4_ in 1 L, diluted ×10). Next, the cells were embedded in 2% semi-solid agar (at 40–45 °C), and after solidification cut into small cell dense pieces. The cells in the agar pieces were subsequently post-fixed in 2% (w/vol) osmium tetroxide (OsO_4_) for 1 h at room temperature, followed by three washes in phosphate buffer. Sequential ethanol dehydration was conducted with 10-min exposures to the following series: 30%, 50%, 70%, 90% (only a 1-min exposure), 95%, and 100% (2 × 10-min exposures) ethanol. The dehydration was followed by 2 × 15-min incubations in 100% acetone. The cells were embedded in Epon and polymerized at 60 °C for 48 h. The material was cut into ultrathin sections (60 nm) using a microtome (Leica Ultracut UCT, Vienna, Austria), placed on Cu grids, and finally post-stained with uranyl acetate. The sections were examined using a Zeiss EM 906 transmission electron microscope (Oberkochen, Germany) operating at 80 kV using the software ITEM 2004. The experiment and embedding were replicated twice.

### 4.7. Statistical Analysis

Most results were investigated by comparing the slopes from linear regression analyses of different treatments with a one-way analysis of variance (ANOVA), followed by pairwise comparisons using Tukey’s HSD or Student’s *t*-tests. In some cases, a two-way ANOVA was conducted to combine the results of replicated studies, using experiment as a block factor. Data in Section 2.2 (Figure 3) and Section 2.2 (Figure 2b) were log and square root transformed, respectively, before analysis. For the amino acid experiment (Section 2.2) and the BMAA stability assay (Section 2.4, Table 1), the ANOVA analyses were followed by pairwise *t*-tests adjusted with Bonferroni correction. The significance level used was 0.05, with correction of *p*-values for multiple pairwise comparisons. The analyses were performed using Stata version 12 or Microsoft Office Excel 2010.

## 5. Conclusions

Despite the potential link between the cyanotoxin BMAA and human neurodegeneration, and given accumulating knowledge on the toxicity of BMAA in eukaryotic cells, the physiological role of BMAA in prokaryotic cyanobacteria remains poorly understood and unexplored. This is the first report on the effects of BMAA on nitrogen fixation in a filamentous, heterocystous cyanobacterium. Although BMAA is produced by *Nostoc* sp. PCC 7120, it acts as a severe inhibitor of a key physiological process, nitrogen fixation, when externally added at micromolar concentrations. BMAA also hampered growth and led to a massive accumulation of glycogen, suggesting that although BMAA is an amino acid with two nitrogen atoms and could thus potentially act as a nitrogen source, BMAA elicits severe nitrogen depletion. Our data, therefore, suggest that other cellular mechanisms related to, for instance, impaired glutamine biosynthesis/degradation or oxidative stress are in effect.

## Supplementary Files

Supplementary File 1 Supplementary Information (PDF, 225 KB)
