# Supplementary material for: BMAA Inhibits Nitrogen Fixation in the Cyanobacterium Nostoc sp. PCC 7120
Source: Mar Drugs. 2013 Aug 21;11(8):3091–108. doi: 10.3390/md11083091 (PMC3766884; doi:10.3390/md11083091)
Supplement: Supplementary File 1 — Supplementary Information (PDF, 225 KB) [file marinedrugs-11-03091-s001.pdf]

Supplementary Information

Figure S1. ESI-LC-MS/MS spectra of BMAA/DAB and BMAA in *Nostoc* sp. PCC 7120. BMAA was detected using the following strict criteria: retention time, diagnostic fragment, and fragment ratio. The BMAA MRM transition used was (459.1 > 119.1) and the diagnostic transition (459.1 > 258.1). DAB was detected using the MRM transition (459.1 > 119.1) and the diagnostic transition (459.1 > 188.1). The bottom row of panels display total ion count (TIC) and the three panels above show the different MRM transitions. Color legends for the MRM transitions: 459.1 > 258.1 (black), 459.1 > 188.1 (purple), 459.1 > 119.1 (green), and TIC *m/z* 459 (red). Numbers at peaks denote: retention time (Rt) (upper) and area (lower). The *y*-axis shows the relative intensity of peaks and the *x*-axis displays retention time (min). (**A**) BMAA/DAB standard demonstrating baseline separation between BMAA (Rt 2.78) and DAB (Rt 2.87). The fragment ratio of *m/z* 119 and *m/z* 258 was 5.3 for the BMAA standard. A 10% variation of this ratio for positive identification of BMAA in a biological sample was allowed. (**B**) The *Nostoc* sp. PCC 7120 sample shows a clearly distinguishable BMAA peak (Rt 2.75, fragment ratio 5.1 with S/N 193) with adequate separation from adjacent peaks. No DAB was detected as the *m/z* 119 fragment corresponding to underivatized DAB was not observed.

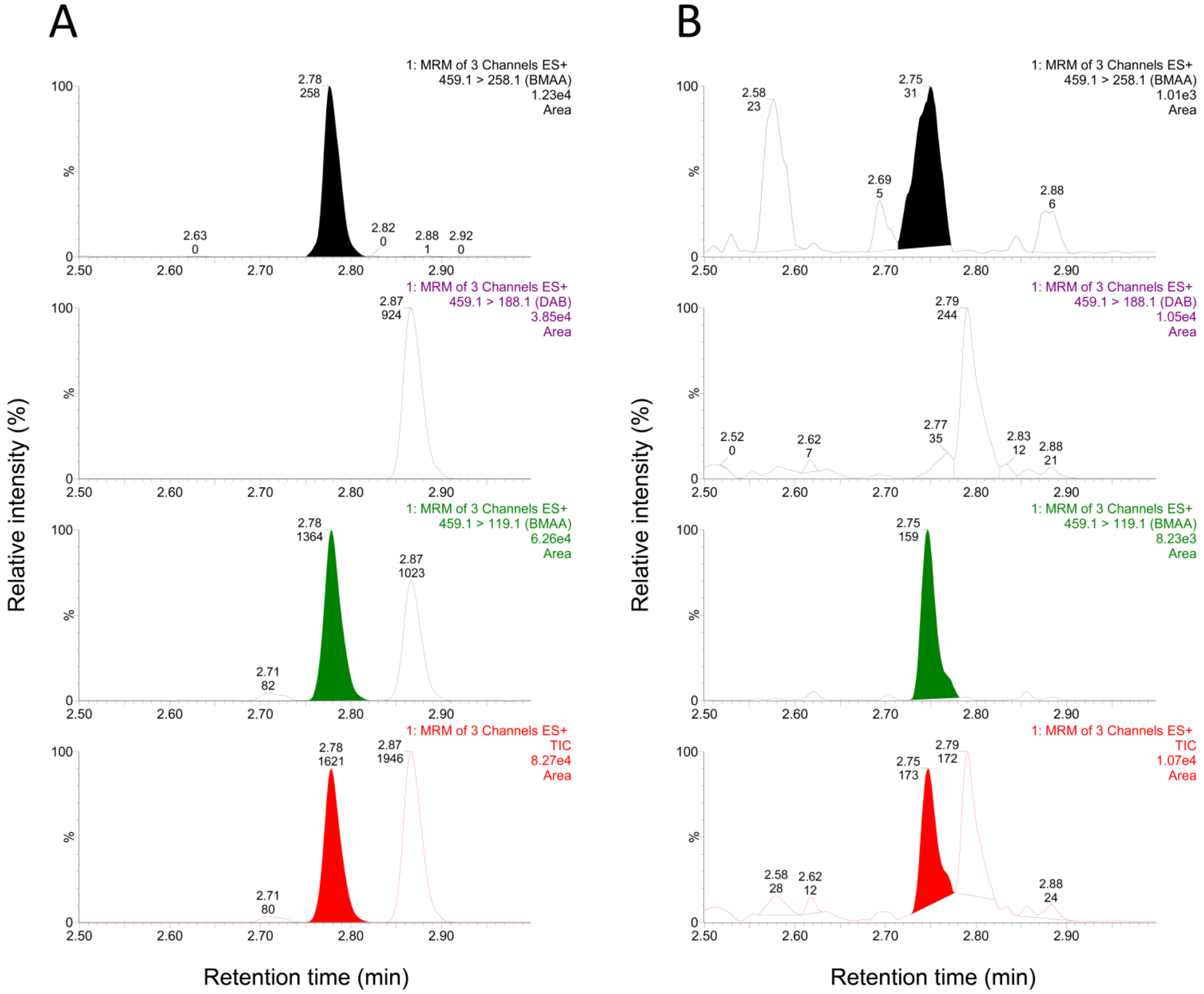

Sample preparation was according to Spáčil *et al.* [1]. Derivatized samples/standards (6-aminoquinolyl-*N*-hydroxysuccinimidyl carbamate, Waters AccQ·Tag Ultra Kit, Waters, Milford, MA, USA) were analyzed on an Acquity UPLC Xevo TQ-MS operated in positive ion mode. Separation was carried out on an Acquity UPLC® BEH C18 column (100 × 2.1 mm, 1.7 µm particle size) with a column temperature of 55.0 °C and a sample injection volume of 10 µL. Eluent A consisted of 0.01% formic acid and 0.05% ammonia in water and eluent B was 0.01% formic acid in methanol. The gradient elution program was as follows: 0.0 min, 0.1% B; 0.54 min, 0.1% B; 4.0 min, 55.0% B; 4.1 min, 100% B; 4.6 min, 100% B; 4.7 min, 0.1% B; and 5.7 min, 0.1% B with a flow rate of 0.6 mL/min. Ion source parameters were: capillary voltage (0.85 V), cone voltage (30 V), source temperature (150 °C), desolvation temperature (550 °C), and desolvation gas flow (1000 L/h).

Reference

1. Spáčil, Z.; Eriksson, J.; Jonasson, S.; Rasmussen, U.; Ilag, L.L.; Bergman, B. Analytical protocol for identification of BMAA and DAB in biological samples. *Analyst* **2010**, *135*, 127–132.

© 2013 by the authors; licensee MDPI, Basel, Switzerland. This article is an open access article distributed under the terms and conditions of the Creative Commons Attribution license (http://creativecommons.org/licenses/by/3.0/).
